# Severe acute respiratory syndrome coronavirus 2 ORF3a induces the expression of *ACE2* in oral and pulmonary epithelial cells and the food supplement Vita Deyun^®^ diminishes this effect

**DOI:** 10.3892/etm.2021.9916

**Published:** 2021-03-16

**Authors:** Adriana Aguilar-Lemarroy, Apolinar López-Uribe, José Sánchez-Corona, Luis Felipe Jave-Suárez

**Affiliations:** Division of Immunology, Western Biomedical Research Center, Mexican Social Security Institute, Guadalajara, Jalisco 44340, Mexico

**Keywords:** severe acute respiratory syndrome coronavirus 2, open reading frame 3a, Vita Deyun, phytonutrients, food supplement, coronavirus disease 2019

## Abstract

The coronavirus disease 2019 (COVID-19) pandemic has become a serious global health problem and numerous studies are currently being conducted to improve understanding of the components of the severe acute respiratory syndrome coronavirus 2 (SARS-CoV-2) virus, as well as to identify solutions that mitigate the effects of COVID-19 symptoms. The nutritional supplement Vita Deyun^®^ is composed of silymarin, glutathione, vitamin C and selenium. Studies of its individual components have demonstrated their benefits as anti-inflammatory agents, antioxidants and enhancers of the immune response. Therefore, the present study aimed to evaluate the *in vitro* effects of Vita Deyun on the expression of angiotensin-converting enzyme 2 (*ACE2*) in diverse cell lines, as well as in the presence or absence of the SARS-CoV-2 open reading frame (ORF)3a protein. Through reverse transcription-quantitative PCR, the use of viral particles containing SARS-CoV-2 ORF3a and bioinformatics analysis via the National Center for Biotechnology Information databases, *ACE2* was determined to be highly expressed in oral and skin epithelial cells, with a lower expression observed in lung cells. Notably, the expression of SARS-CoV-2 ORF3a increased the level of *ACE2* expression and Vita Deyun treatment diminished this effect. In addition, Vita Deyun treatment markedly decreased interleukin-18 mRNA levels. The combination of phytonutrients in Vita Deyun may help to boost the immune system and could reduce the effects of COVID-19. Ongoing clinical studies are required to provide evidence of the efficacy of Vita Deyun.

## Introduction

The current coronavirus disease 2019 (COVID-19) pandemic emerged in late 2019 in Wuhan, China and has spread rapidly throughout the world. It is estimated that 20% of individuals infected with the causative viral agent, severe acute respiratory syndrome coronavirus 2 (SARS-CoV-2), develop COVID-19 disease, which is causing the collapse and saturation of health systems in several countries (1). The approximate fatality rate of the disease ranges from 4 to 10% and the associated symptoms include high fever, dry cough, tiredness, sore throat, diarrhea, conjunctivitis, smell or taste loss and, in more serious conditions, difficulty breathing and the inability to speak and move (2). Severe disease is related to the presence of comorbidities, such as hypertension, diabetes, cardiovascular diseases and respiratory diseases (3). Mexico, in particular, has a high rate of hypertension, obesity and diabetes, placing its population in an unfavorable condition to deal with the pandemic (4).

SARS-CoV-2 is a single-stranded RNA virus ~65-125 nm in diameter, which can be transmitted by air or by contact with contaminated surfaces. The main routes of entry into the body are the nasal, oral or ocular routes (5). Once the virus enters the body, it will infect mucosa, in particular the nasal and laryngeal mucosa (6). As the infection progresses, it travels to the lungs through the respiratory tract (7). From the lungs, the virus can enter the peripheral blood, leading to viremia and the attack of other organs (8). The host receptor protein used by the virus to infect cells is angiotensin-converting enzyme 2 (ACE2) (9,10) and expression of this receptor has been observed in various tissues, such as the heart, liver, testicles, kidneys and intestines. Typically, the ACE2 protein is responsible for the regulation of heart and kidney function, as well as the control of blood pressure (11).

Among the 14 open reading frames (ORFs) identified in the SARS-CoV-2 genome, ORF3a codes for 274 of the amino acid proteins proposed to be responsible for its high virulence in humans (12). It has been described to be similar to ion channel proteins that can help to create a more permeable membrane in infected cells (13). ORF3a presents a plethora of activities, among which are the reduction of type I interferon (IFN) production, fibrinogen induction, production and release of cytokines, activation of the NF-κB pathway, production of chemokines, Golgi reticulum fragmentation, endoplasmic reticulum stress, accumulation of intracellular vesicles and cell death (14). All of these characteristics identify ORF3a as an ideal therapeutic target in the fight against COVID-19.

Vita Deyun^®^ is a solution based on 1 mg silymarin, 1 mg glutathione (GSH), 1 mg vitamin C and 0.050 mg selenium (https://vitadeyun.com.mx). The individual components of this solution have been extensively investigated for their effects on different biological aspects of cells and organisms, and they have demonstrated benefits as anti-inflammatory agents, antioxidants and enhancers of the immune response, as described in detail below.

Silymarin is a natural compound derived from the plant milk thistle (*Silybum marianum*) and it has been traditionally used to treat hepatological conditions (15). Evidence of its beneficial uses range from antioxidant and anti-inflammatory to immunomodulatory activities (16-19). In addition, it has been shown that silymarin has a low toxicity of ≤40 µg/kg weight and ≤425 µg/ml in cell culture (20). Furthermore, a protective effect during lung injury has been demonstrated through decreasing the infiltration of inflammatory cells, suppressing the activity of myeloperoxidase, decreasing the production of nitric oxide and the activity of nitric oxide synthase, and reducing the protein levels of pro-inflammatory mediators, superoxide dismutase, catalase and GSH peroxidase (21).

GSH is an antioxidant comprising three amino acids, glutamine, glycine and cysteine, making it a small molecule with low molecular weight. GSH is normally produced in hepatocytes and exported into circulation to be transported for use in other cells; moreover, GSH in plasma is maintained at a very low concentration because cell uptake by numerous other cells is rapid (22,23). This process requires the action of gamma-glutamyl transpeptidase, which is commonly found on the surface of cells (24). There is evidence that GSH deficiency leads to increased levels of oxidative stress. Indeed, a protective role of GSH has been demonstrated against inflammatory lung diseases (25-27). GSH is essential for numerous innate and adaptive immune system functions, including proliferation of T lymphocytes (28,29), phagocytic activity of neutrophils (30), dendritic cell functions (31) and presentation of antigens by antigen-presenting cells (32). One of the first steps in antigen degradation and processing is the reduction of disulfide bonds, which requires GSH (33). Although, GSH inhibits the production of certain inflammatory cytokines, it is necessary that dendritic cells maintain an adequate level of IFN-γ (34), an essential condition for host defense against intracellular pathogens. There are >650 studies registered in ClinicalTrials.gov (www.clinicaltrials.gov, accessed: 30 December 2020) claiming to demonstrate the various benefits of using GSH.

Regarding ascorbic acid, there is ample evidence from *in vitro* studies, animal experiments and clinical trials that vitamin C might exert an antiviral effect (35,36). Based on this finding and since, to the best of our knowledge, no specific antiviral drug has yet been proven to be truly effective against COVID-19, the supplementation of micronutrients, such as vitamin C has become relevant in the management of COVID-19, as it might improve the immunological response against SARS-CoV-2.

Viral infections are characterized by deficient micronutrient stores, particularly vitamins and trace elements, such as zinc, iron, selenium, magnesium and copper. Lifestyle and appropriate nutrition may offer further antiviral approaches for public health (37). Selenium, one of the compounds of Vita Deyun, is a natural trace element essential to human health and has a key and complex role in the immune system (38). Selenium has a history of reducing the incidence and severity of various viral infections; it is well-documented that selenium deficiency is associated with higher susceptibility to RNA viral infections and more severe disease outcomes (39).

As described above, the individual components of Vita Deyun have been proven to have beneficial effects in viral and respiratory diseases, which supports the proposal for evaluation of the effect of Vita Deyun on SARS-CoV-2 ORF3a. Therefore, the aim of the present study was to evaluate the *in vitro* effect of Vita Deyun solution on the expression of *ACE2* in diverse cell lines, particularly in the presence of SARS-CoV-2 ORF3a expression.

## Materials and methods

### 

#### Cell culture and treatment

HaCaT, DOK, A549, H1299 and Lenti-X 293T cells were cultured in DMEM medium supplemented with 10% fetal bovine serum, 100 U/ml penicillin, and 1 mg/ml streptomycin. The cultures were maintained at 37˚C in a 5% CO_2_ atmosphere. The aforementioned products were acquired from Gibco^®^ (Thermo Fisher Scientific, Inc.). HaCaT cells are human keratinocytes spontaneously transformed from histologically normal skin and were obtained from Dr Petra Boukamp (German Cancer Research Center) (40). The DOK cells are human dysplastic oral keratinocytes (from the tongue) and were provided by the cell bank of the Center for Scientific Instrumentation of the University of Granada (41). The A549 and H1299 cell lines are derived from lung cancer tissues and were acquired from the ATCC. Lenti-X 293T cells were obtained from Clontech Laboratories, Inc. Certain cell lines underwent additional authentication using the Multiplex human Cell Authentication test performed by Multiplexion GmbH (www.multiplexion.de).

Vita Deyun solution (Envasadora D´Aqua, S.A de C.V.) contains 1 mg silymarin, 1 mg GSH, 0.05 mg Selenium, 1 mg ascorbic acid and 0.1 mg sodium benzoate in 100 ml water. For the treatment, 3x10^6^ cells were seeded on p100 petri dishes in 9.5 ml complete DMEM and left for 24 h. They were then treated with 500 µl Vita Deyun solution for 4 h and used for RNA isolation. Cells were cultivated at 37˚C in a 5% CO_2_ atmosphere, unless otherwise stated. Cells in culture were continually monitored microscopically when seeding them or before and after a treatment by using an inverted microscope Primovert with a x400 magnification (ZEISS company).

#### RNA extraction and reverse transcription-quantitative PCR (RT-qPCR)

For RNA isolation (total RNA), the GeneJet RNA Purification Kit (Thermo Fisher Scientific, Inc.) was used according to the manufacturer's protocol. Thereafter, to obtain cDNA, the Transcriptor First Strand cDNA Synthesis Kit (Roche Applied Science) was utilized. Briefly, 5 µg total RNA was primed with oligo(dT) and reverse transcribed as recommended by the manufacturer's instructions. Specific primers for all analyzed genes were engineered by utilizing Oligo v.6.0 software (Molecular Biology Insights, Inc.) and sequences were obtained from the National Center of Biotechnology Information (NCBI). Ribosomal protein L32 (RPL32) and ribosomal protein S18 (RPS18) served as reference genes in the present study. The primer sequences are presented in Table I. For qPCR reactions, the Light Cycler 2.0 system and the LightCycler FastStart DNA Master PLUS SYBR-Green I Kit (Roche Applied Science) were used. The thermocycling conditions were as follows: A first denaturalization step of 10 min at 95˚C, followed by 40 cycling steps of 10 sec at 95˚C, 10 sec using the temperature shown for each primer pair in Table I, and 12 sec at 72˚C. Expression levels were normalized using the expression of the reference genes as internal controls. The expression of the control groups was set as the reference value and the fold-change variations in gene expression were calculated using the Pfaffl mathematical model (42).

#### Lentivirus transfection, transduction and cell line establishment

Viral particles were produced using Lenti-X 293T cells. Briefly, 3x10^6^ Lenti-X-293T cells were seeded one day before transfection. For the transfection assays, Lipofectamine^®^ 3000 (Invitrogen; Thermo Fisher Scientific, Inc.) was used according to the manufacturer's protocol and utilizing a mix of Lenti-X HT packaging systems (4 µg) and 1 µg of one of the following lentiviral vectors, pLVX-Puro (Takara Bio, Inc.) or the pLVX-EF1alpha-SARS-CoV-2-ORF3a-2xStrep-IRES-Puro vector (cat no. 141383; Addgene, Inc.) (43). After 48 h, to eliminate detached cells and cell detritus, the medium containing the viral particles was filtered through a 0.45-µm polyethersulfone membrane (Merck KGaA). The titer of viral particles was tested using Lenti-X GoStix (Takara Bio, Inc.). The medium containing the viral particles was aliquoted and stored at -80˚C until use. For the transduction assays, 1x10^6^ target cells were seeded one day before transduction. The target cells were transduced with 200 µl medium containing lentiviral particles (~1x10^6^ Inclusion-Forming Units). To obtain stable cell lines, after 72 h of transduction, the cells were incubated with 0.5 µg/ml puromycin for 3 weeks. The medium containing puromycin was replaced every three days; stable cells were cultured under normal conditions without puromycin. Cells were always cultivated or treated at 37˚C in a 5% CO_2_ atmosphere.

#### Statistical analysis

Data were obtained from three independent experiments with paired observations (each experiment was performed simultaneously and in duplicate). The data are presented as the mean ± standard deviation. The one-way ANOVA test for multiple comparisons was employed to compare data from the different treatments. The Bonferroni test was used as post hoc test and P<0.05 was considered to indicate a statistically significant difference. Statistical analysis was performed using the GraphPad prism v.5 software (GraphPad Software, Inc.).

## Results

### 

#### ACE2 is highly expressed in oral and skin epithelial cells and has low expression in lung cells

**Figure 1:**
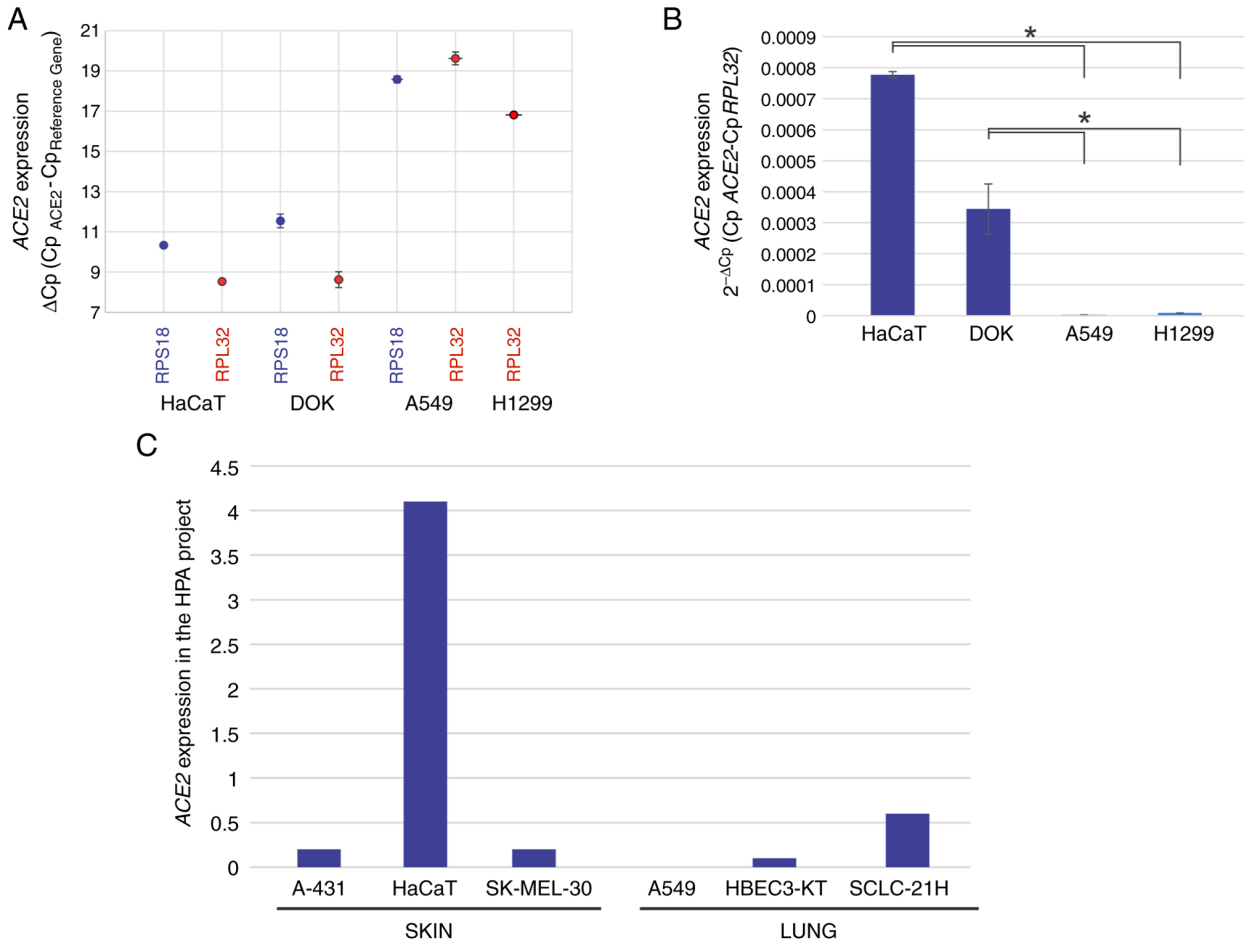
Reverse transcription-quantitative PCR was performed to analyze expression of *ACE2* in oral-, skin-, and lung-derived epithelial cells. (A) *ACE2* expression in HaCaT (skin), DOK (oral) and A549 and H1299 (lung) cells was calculated as ΔC_p_ (values inversely proportional to expression) or (B) calculated using the 2^-ΔCp^ algorithm (mRNA quantity values). The values are presented as the mean ± SD from at least three different experiments, with *RPL32* and *RPS18* serving as reference genes. ^*^P<0.05. (C) *ACE2* expression in skin- and lung-derived epithelial cells using data from the HPA project. The values represent an estimation of the abundance of *ACE2* transcripts in each cell line. Data are derived from RNA-seq experiments and a value of 1.0 is defined as the threshold for *ACE2* expression at the protein level. *ACE2*, angiotensin-converting enzyme 2; HPA, Human Protein Atlas.

With the aim of achieving a suitable study model that would allow for evaluation of the effects of Vita Deyun solution on the expression of *ACE2* and pro-inflammatory cytokines, the lung-derived A549 and H1299 cell lines, the oral epithelial DOK cell line and the skin-derived HaCaT cell line were analyzed for the expression of *ACE2* using RT-qPCR. As presented in Fig. 1A, the ΔC_p_ values for *ACE2* were 8-12 in HaCaT and DOK cells, whereas in A549 and H1229 cells, the ΔC_p_ values were 17-20. As ΔC_p_ values represent an inverse correlation to expression, a marked decrease in *ACE2* levels was determined in the lung-derived cell lines. For improved interpretation of these results, the data are presented as 2^-ΔCp^ values (mRNA expression density), as shown in Fig. 1B. In addition, to corroborate these results, data regarding the reported expression of *ACE2* was obtained from the Human Protein Atlas (HPA) project version 20.1 (https://www.proteinatlas.org/ENSG00000130234-ACE2/summary/rna), where information regarding RNAseq gene expression in different cell lines is available (RNA summary-Normalized-HPA cell lines). As presented in Fig. 1C, the expression of *ACE2* was high in HaCaT cells, but not in the skin-derived A-431 and SK-MEL-30 cell lines included in the HPA project. By contrast, the lung-derived HBEC3-KT and SCLC-21H cells exhibited generally low expression of *ACE2* and very low expression was also observed in the A549 cells.

#### SARS-CoV-2 ORF3a modulates ACE2 expression in oral- and lung-derived epithelial cells and this effect was hampered by Vita Deyun treatment

**Figure 2:**
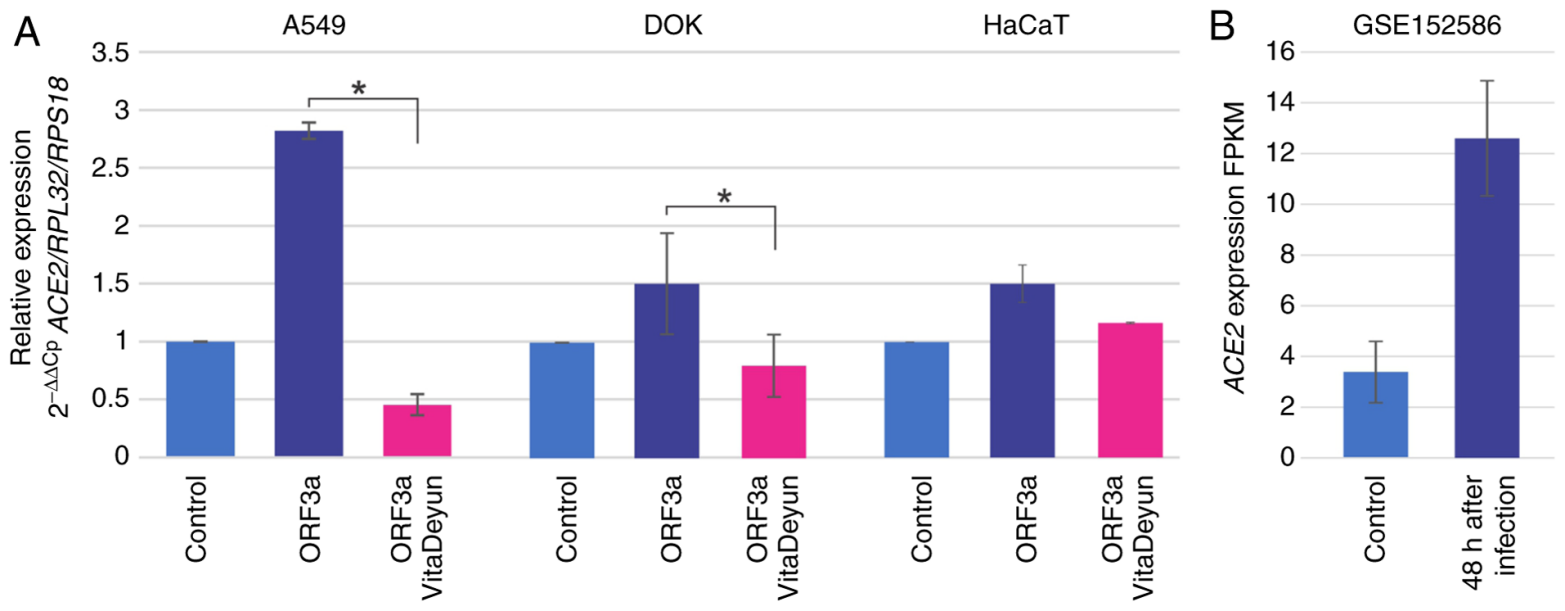
Relative *ACE2* expression in cells expressing SARS-CoV-2 ORF3a. (A) A549, DOK and HaCaT cells were transduced for 24 h with lentiviral particles carrying ORF3a from SARS-CoV-2 and were subsequently treated with Vita Deyun solution. The expression of *ACE2* was determined by reverse transcription-quantitative PCR, with *RPL32* and *RPS18* serving as reference genes and the value of untreated cells as the control (2^-ΔΔCp^ algorithm). The values are presented as the mean ± SD from at least three different experiments. ^*^P<0.05. (B) Data from project GSE152586 of the Gene Expression Omnibus were used to determine variations in *ACE2* expression following 48 h of SARS-CoV-2 infection in alveolar spheroids. The values are expressed in fragments per kilobase of exon model per million reads mapped (FPKM), which is a normalized estimation of gene expression based on RNA-seq data. *ACE2*, angiotensin-converting enzyme 2; SARS-CoV-2, severe acute respiratory syndrome coronavirus 2; *ORF3a*, open reading frame 3a; *RP*, ribosomal protein.

To analyze the influence of ORF3a expression in oral- and lung-derived epithelial cells, DOK and A549 cells were transduced with lentivirus carrying ORF3a of SARS-CoV-2. HaCaT cells were included in the present study due to their high levels of *ACE2* mRNA expression. ORF3a induced an increment in *ACE2* mRNA levels in all of the analyzed cells, most notably in the lung-derived A549 cells, where the expression was increased by almost 3-fold (Fig. 2A). The cells were treated for 24 h with the food supplement Vita Deyun to evaluate whether it modulated the effect of ORF3a on *ACE2*, and the expression levels of *ACE2* were determined by RT-qPCR. Notably, it was observed that Vita Deyun treatment markedly inhibited the expression of *ACE2* induced by ORF3a and, in certain cases, the expression levels of *ACE2* were even lower than the basal levels of the control (untreated) cells (Fig. 2A). Morphologically, no change or adverse effect on the growth of the cells was observed in the presence of Vita Deyun when compared to the untreated cells (data not shown). The data from the GSE152586 study of the Gene Expression Omnibus from NCBI (https://www.ncbi.nlm.nih.gov/geo/) was analyzed to further support the present findings. In the GSE152586 study, human-derived alveolar organoids were infected with SARS-CoV-2 and, following 48 h of infection, gene expression was evaluated using RNAseq (44). The data showed an evident increase in *ACE2* expression following infection (Fig. 2B).

#### Differential responses in cytokine expression following Vita Deyun treatment of epithelial cells constitutively expressing ORF3a

**Figure 3:**
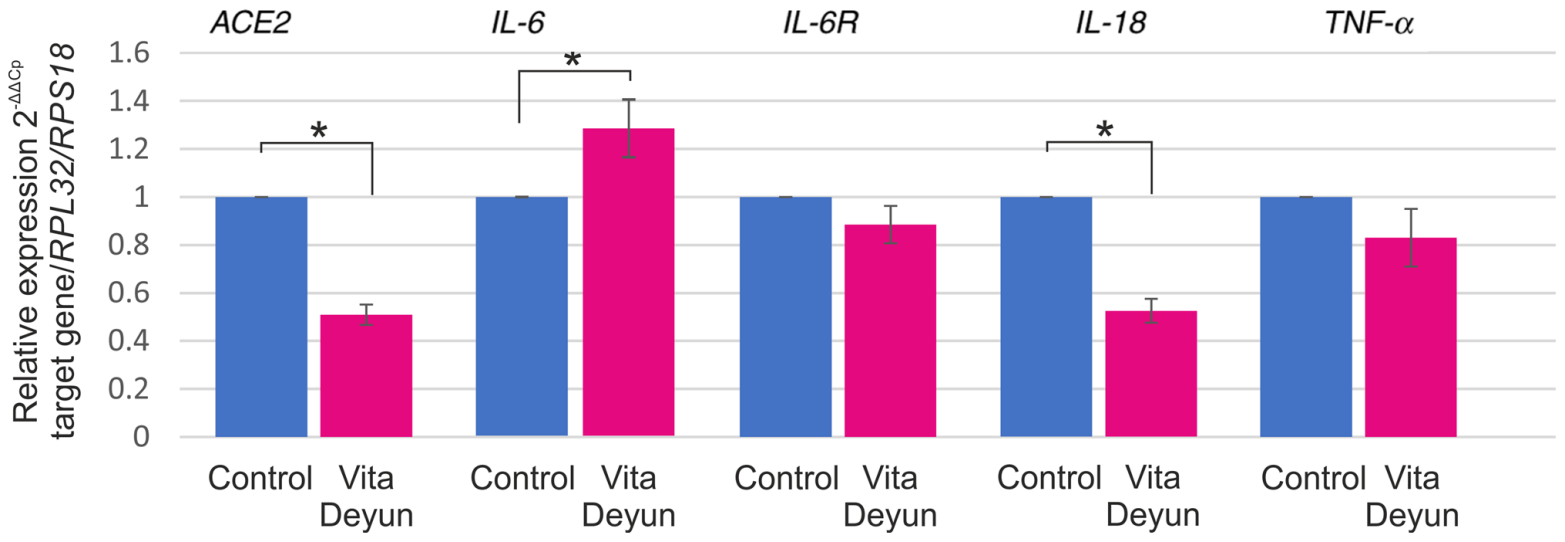
Cytokine mRNA levels in A549 cells constitutively expressing SARS-CoV-2 ORF3a. A model of A549 cells that constitutively expressed the ORF3a were developed and analyzed to determine variations in the expression of *ACE2*, *IL-6*, *IL-6R*, *IL-18* and *TNF-α* by reverse transcription-quantitative PCR. The expression is presented as relative expression and was determined using the 2^-ΔΔCp^ algorithm with *RPL32* and *RPS18* serving as the reference genes. The values are presented as the mean ± SD from at least three different experiments. ^*^P<0.05. SARS-CoV-2, severe acute respiratory syndrome coronavirus 2; ORF3a, open reading frame 3a; *ACE2*, angiotensin-converting enzyme 2; *IL*, interleukin; *TNF*-α, tumor necrosis factor-α; *RP*, ribosomal protein.

In addition to its effect on *ACE2* expression, the effect of Vita Deyun on the expression of interleukin (IL)-6, IL-6 receptor (IL-6R), IL-18 and tumor necrosis factor (TNF)-α was evaluated. A model of A549 cells that constitutively expresses ORF3a from SARS-CoV-2 was established. Lentiviral particles carrying ORF3a and a puromycin resistance sequence were generated and used to transduce A549 cells. The generation of this model was particularly difficult, since ORF3a expression was toxic for the cells and a high percentage of dead cells was microscopically observed (data not shown). Following selection with puromycin, only a few clones were obtained, and the ORF3a-positive cells were observed to have a lower proliferation rate (data not shown). Furthermore, DOK and HaCaT cells were transduced with ORF3a, but the cells did not survive. The A549 ORF3a-positive cells were treated with Vita Deyun and the expression of *ACE2* and the aforementioned cytokines was evaluated. As shown in Fig. 3, a significant decrease in the *ACE2* mRNA levels was observed. Whereas a slight increase in *IL-6* mRNA expression was observed. Slight decreases were observed in *IL-6R* and *TNF-α* and a considerable downregulation of *IL-18* was observed.

## Discussion

Sufficient evidence exists to indicate that ACE2 is the main host cell receptor of SARS-CoV-2 for entry into cells to cause infection. *ACE2* expression in oral-, skin- and lung-derived epithelial cells was observed in the present study. DOK and HaCaT cells were found to express higher levels of *ACE2* than the analyzed lung-derived cells. Xu *et al* (45) also reported high levels of ACE2 receptor on the epithelial cells of oral mucosa.

A notable observation that was determined in the present study is that the SARS-CoV-2 ORF3a protein induces the expression of *ACE2*. This observation was demonstrated using two different methodological strategies, as shown in Figs. 2 and 3. To the best of our knowledge, the present study is the first to demonstrate this effect. The lack of studies could be partly due to the fact that ORF3a is harmful to cells; as mentioned in the results section of the present study, only stably transformed A549 cells constitutively expressing ORF3a were obtained and similar attempts were not successful using HaCaT or DOK cell lines due to cell lethality. Recently, Ren *et al* (46) demonstrated that ORF3a of SARS-CoV-2 induces apoptosis in cells.

Moreover, the present study focused on determining whether the food supplement Vita Deyun is capable of regulating the expression of *ACE2*, the receptor reported to be recognized by SARS-CoV-2(47). As hypothesized, Vita Deyun reduced the expression of *ACE2* in various cell models (Fig. 2).

One of the components of the dietary supplement Vita Deyun is silymarin, for which there is evidence of antiviral properties. Silymarin has been observed to hijack the replication system of the chikungunya virus, reducing viral replication and decreasing the production of viral proteins (16-19). Silymarin also inhibits the entry of hepatitis C virus into its target cells and, in infected cells, it inhibits the expression of viral RNA and the production of viral proteins (48). To the best of our knowledge, there are currently no clinical reports that involve the use of silymarin against COVID-19; however, there are studies that suggest its potential use to combat this disease, as mentioned below. Taking into account the biophysical and structural evidence that spike proteins possess high binding affinity toward host ACE2 and the viral hemagglutinin esterase (HE) glycoprotein receptor, Patel *et al* (49) used a combination of various computational approaches to identify potential inhibitors of HE by employing the naturally occurring plant-based anticancer compound-activity-target database (http://crdd.osdd.net/raghava/npact/). Subsequently, the best-scoring molecules were validated using molecular dynamics simulations and the authors identified silymarin (among other compounds) as a potential HE inhibitor with improved binding energy. Furthermore, Kumar *et al* (50) demonstrated that the binding of phytochemicals, such as sarsasapogenin, ursonic acid, curcumin, ajmalicine, novobiocin and silymarin to the SARS-CoV-2 nonstructural protein 15 protein might play a key role in inhibiting SARS-CoV-2 replication. In addition, Gorla *et al* (51) screened essential flavonoids against possible protein targets, such as the SARS-CoV-2 spike glycoprotein receptor-binding domain of the S protein (RBD-S) and the host ACE2 protease domain (PD-ACE-2) using *in silico* molecular docking studies and determined that biochanin A and silymarin bind significantly at the active sites of RBD-S and PD-ACE-2.

Another component of Vita Deyun is GSH. Kim *et al* (52) applied advanced bioinformatics computational approaches to identify which existing Food and Drug Administration-approved drugs could block coronaviruses from entering cells by binding to ACE2 or transmembrane protease, serine 2, or attenuate the expression of genes induced by coronaviruses; the authors identified various ACE inhibitors, a β-lactam antibiotic, two antiviral agents (Fosamprenavir and Emricasan) and GSH. A common denominator in COVID-19 patients appears to be impaired redox homeostasis, which is responsible for the accumulation of reactive oxygen species. GSH levels could be critical in extinguishing the exacerbated inflammation observed in COVID-19 cases. Therefore, it was proposed by Silvagno *et al* (53) that restoring GSH levels in patients with severe symptoms of COVID-19 may be a therapeutic alternative to prevent and control the disease. In agreement with this, Guloyan *et al* (54) suggested that the use of liposomal GSH could be beneficial in COVID-19 patients.

Regarding selenium, Taylor and Radding (55) mentioned that the resulting collateral damage due to increased oxidative stress and inflammation would be exacerbated by dietary deficiency of selenium and GSH precursors. In the review by Hiffler and Rakotoambinina (39), it was observed that low-selenium status is common in individuals considered at risk of developing severe COVID-19, particularly in the elderly. In a study conducted by Moghaddam *et al* (56) in Germany, it was shown that serum selenium levels were highly correlated with COVID-19 outcome in hospitalized patients and the authors determined that selenium deficiency is associated with mortality risk from COVID-19.

Vitamin C has been reported to have multiple pharmacological characteristics, including antiviral, antioxidant, anti-inflammatory and immunomodulatory effects, which establish it as a potential therapeutic option in the management of COVID-19 (57,58). Indeed, previous studies have shown promising results when intravenously applying high doses of vitamin C for the management of COVID-19 (59,60). In accordance with these studies, Feyaerts and Luyten (61) proposed that using a relatively low dose of vitamin C as a prophylaxis, or an intravenous-based high-dose regimen in cases of severe COVID-19, may be beneficial. Similarly, Carr and Rowe (62) asserted that, due to its excellent safety profile, low cost and potential for rapid upscaling of production, the administration of vitamin C to patients with hypovitaminosis C and severe respiratory infections, such as COVID-19, appears warranted.

Regarding the downregulation of *IL-18* and *TNF-α* induced by Vita Deyun (as presented in Fig. 3), it has been recently reported that use of curcumin and silymarin alone or in combination for treatment of gamma ray-induced nephrotoxicity in rats resulted in a marked decline in the serum levels of the two cytokines (63).

Currently, there are various drugs and vaccines being evaluated for their safety, efficacy and effect against COVID-19, although a significant period of time will be required for their validation. Therefore, it is also important to consider the use of natural components that have no side effects in humans as alternative tools to combat COVID-19. Numerous nutraceuticals have a proven ability to boost the immune system in addition to having antiviral, antioxidant and anti-inflammatory effects (64). However, ongoing clinical trials are expected to provide more definitive evidence.

Vita Deyun solution is a combination of various phytonutrients in the form of a food supplement that, based on the current *in vitro* results and the abovementioned evidence, may help to boost the immune system and prevent virus spread, thereby potentially reducing the symptoms of COVID-19. However, it is clear that clinical trials are required to fully determine its efficacy. Currently, clinical protocols that have already been authorized by ethics committees are being conducted in various states of Mexico to determine the efficacy of Vita Deyun against the diverse symptoms associated with COVID-19.

## Figures and Tables

**Table I:** Primers used for reverse transcription-quantitative PCR amplification.

	Primers (5'-3')			
Gene	Forward	Reverse	Product length (bp)	Temperature (˚C)
*ACE2*	TTCTTTGTCACTGCACCTAA	AATGCCAACCACTATCACTC	225	58
*IL-6*	TACAAAAGTCCTGATCCAGTTC	AAGAAGGAATGCCCATTAAC	245	60
*IL-6R*	GGCACGCCTTGGACAGAATCC	CCGCAGCTTCCACGTCTTCTT	252	62
*IL-18*	CCCCGGACCATATTTATTATA	CATGTCCTGGGACACTTCT	202	58
*TNF-α*	CCTGTAGCCCATGTTGTAGCA	GCCTTGGCCCTTGAAGAG	171	62
*RPL32*	GCATTGACAACAGGGTTCGTAG	ATTTAAACAGAAAACGTGCACA	320	60
*RPS18*	CGATGGGCGGCGGAAAA	CAGTCGCTCCAGGTCTTCACGG	283	60

*ACE2*, angiotensin-converting enzyme 2; IL, interleukin; R, receptor; *TNF-*α, tumor necrosis factor-α; RP, ribosomal protein.

## Data Availability

The datasets used and/or analyzed during the current study are available from the corresponding author on reasonable request.
